# Survival Outcomes of Patients with Follicular Lymphoma after Relapse or Progression: A Single-Center Real-World Data Analysis

**DOI:** 10.1155/2022/2263217

**Published:** 2022-09-26

**Authors:** Yong-Pyo Lee, Min-Sang Lee, Sang Eun Yoon, Junhun Cho, Yeong Hak Bang, Joon Ho Shim, Won Seog Kim, Seok Jin Kim

**Affiliations:** ^1^Division of Hematology-Oncology, Department of Medicine, Samsung Changwon Hospital, Sungkyunkwan University School of Medicine, Changwon, Republic of Korea; ^2^Division of Oncology/Hematology, Department of Internal Medicine, Soonchunhyang University Hospital Cheonan, Suncheonhyang 6-Gil, Dongnam-gu, Cheonan-si, Chungcheongnam-Do, Republic of Korea; ^3^Division of Hematology-Oncology, Department of Medicine, Samsung Medical Center, Sungkyunkwan University School of Medicine, Seoul, Republic of Korea; ^4^Department of Pathology, Samsung Medical Center, Sungkyunkwan University School of Medicine, Seoul, Republic of Korea; ^5^Department of Digital Health, Samsung Advanced Institute for Health Sciences and Technology, Sungkyunkwan University, Seoul, Republic of Korea; ^6^Department of Dermatology, Samsung Medical Center, Sungkyunkwan University School of Medicine, Seoul, Republic of Korea; ^7^Department of Health Sciences and Technology, Samsung Advanced Institute for Health Sciences and Technology, Sungkyunkwan University, Seoul, Republic of Korea

## Abstract

**Background:**

Follicular lymphoma (FL) is considered incurable because remission and relapse are common. Although various salvage treatment options have been proposed, there is no consensus on treatment strategy for FL patients who failed primary treatment.

**Methods:**

This single-center study analyzed postevent overall survival (OS) among 70 patients who experienced relapse or progression after rituximab-containing immunochemotherapy according to type of salvage treatment and nature of relapse or progression.

**Results:**

Of 70 patients, 42 experienced progression of disease within 24 months (POD24), and six showed disease progression during first-line treatment. Large-cell transformation was found in nine patients with POD24. At the median follow-up of 104 months (95% CI: 90-118 months), POD24 patients experienced significantly worse OS than patients without POD24, and postevent OS was not satisfactory after conventional salvage chemotherapy because the majority of patients relapsed or progressed. However, autologous stem cell transplantation (ASCT) after the first relapse resulted in survival prolongation in patients with POD24. Half of the patients (34/67, 51%) participated in at least one clinical trial during treatment after first relapse, and patients participating in at least one clinical trial irrespective of line of treatment tended to experience better survival.

**Conclusions:**

Relapsed or refractory FL patients showed various clinical courses and treatment outcomes according to relapse or progression. Consolidation treatment with ASCT and active participation to clinical trials might prolong survival duration, especially in POD24 cases.

## 1. Introduction

Follicular lymphoma (FL) is the second most frequent subtype of lymphoma in Western populations, accounting for 35% of all non-Hodgkin lymphomas (NHLs), and is the most common indolent NHL, representing 20%-30% of all cases [1, 2]. For stage I or contiguous stage II patients with FL grade 1/2, involved-site radiotherapy is recommended with curative intent. However, most patients with FL present as stage III or IV, for which there are no established treatment strategies with curative intent [3]. This is why current treatment guidelines recommend either a “watch and wait” approach for patients with no indications for initiating treatment or encourage participation in clinical trials for patients who are eligible, even with newly diagnosed FL. For patients requiring treatment after diagnosis for conditions such as cytopenia, threatened end-organ function, or symptomatic disease progression, immunochemotherapy has been used in clinical practice because CD20-targeting monoclonal antibodies such as rituximab and obinutuzumab have shown promising outcomes in combination with cytotoxic chemotherapies. Thus, immunochemotherapies such as bendamustine plus rituximab (BR) or rituximab plus cyclophosphamide, vincristine, and prednisone with or without doxorubicin (RCHOP or RCVP) are the standard of care for frontline induction treatment for symptomatic, advanced-stage FL patients based on extensive data supporting their efficacy in FL patients [4–9]. However, despite the availability of potent frontline treatments, high probability of recurrence including late relapse persists, and prolonged B-cell depletion caused by rituximab could increase the risk of infectious complications [10, 11].

Accordingly, FL is considered a hard-to-cure disease because patients with FL often live longer than those with aggressive B-cell lymphoma due to its indolent nature but eventually die due to disease progression. Additionally, not all patients follow an indolent clinical course, and early disease relapse accompanied by progression of disease within 24 months (POD24) or relapsed disease with large-cell transformation could have aggressive outcomes [12–14]. Various treatment options have been proposed as salvage treatments for FL patients with relapsed or refractory disease. These include anti-CD20 targeted immunotherapy [15], immunochemotherapy [16–18] followed by autologous stem cell transplantation (ASCT) [19], and chemotherapy-free alternatives [20]. Allogeneic stem cell transplantation is also considered a treatment option [21] but is mostly performed in selected patients who are young and fit. However, there has been no consensus on treatment strategies for FL patients who have failed primary treatment, and their outcomes vary depending on the nature of the disease and type of salvage treatment. Therefore, we analyzed the postevent survival outcomes of FL patients who experienced relapse or progression after first-line treatment with BR, RCHOP, or RCVP to address the impacts of subsequent therapies on survival outcomes.

## 2. Methods

### 2.1. Patients

In this study, we retrospectively analyzed the survival outcomes of FL patients who experienced any relapse, progression, or any cause of death after primary treatment. The study population was collected from two prospective cohort studies (2008-2011, NCT#00822731 and 2012-2016, NCT#01877109) and the lymphoma registry of Samsung Medical Center between March 2003 and February 2019. The selection criteria are as follows: (1) biopsy-proven grade 1, 2, or 3A FL; (2) received primary systemic immunochemotherapy for treatment-requiring conditions, such as B symptoms, bulky disease, rapid progressive disease, or advanced-stage disease; (3) experienced an event during or after primary treatment; 4) relapse or progression was determined by computed tomography (CT) scan or positron-emission tomography/CT scan according to the Lugano classification [22]. Thus, we excluded patients who were observed after diagnosis without treatment. Salvage treatment strategies were classified as participation in clinical trials or conventional treatment with cytotoxic chemotherapy with or without CD20 targeting antibody combination. Clinical trials included all experimental treatments using novel agents or new drugs (either alone or in combination with other treatments), and that were conducted in our institution for relapsed or refractory FL patients after provision of informed consent. Conventional chemotherapies were defined as all salvage treatments consisting of cytotoxic chemotherapies, whereas CD20 targeting antibody-containing immunochemotherapies included combination chemotherapies with CD20 targeting antibodies such as rituximab.

### 2.2. Analysis

The objective of this study was to compare postevent survival outcomes according to subsequent salvage treatments. Overall survival (OS) was measured from the date of diagnosis to the date of death by any cause or last follow-up date, whereas postevent OS was measured from the date of first event to the date of death by any cause, and time to next treatment was measured from the date of first event to the date of a second event requiring any type of salvage treatment. To evaluate parameters that could influence outcomes, clinical and laboratory characteristics reflecting disease burden and aggressiveness were collected including Ann Arbor stage, follicular lymphoma international prognostic index (FLIPI) score, number of nodal involvements, bone marrow involvement, and serum lactate dehydrogenase (LDH). Viral marker information was also collected at the time of registration, such as hepatitis B surface antigen (HBsAg) and hepatitis B core antibody (anti-HBc antibody). POD24 was defined as relapse or progression after the first date of primary first-line treatment. As an exploratory analysis, we also compared mutation profiles of tumor tissue at diagnosis with those of rebiopsy tumor tissue after relapse using archived targeted sequencing data that were collected with informed consent as previously described [23, 24]. Mean sequencing coverage was greater than 700×, and somatic alterations were called by a previously described pipeline: MuTect version 1.1.6, Lowfreq version 0.6.1, Pindel version 0.2.5a4 software, and a custom-built in-house algorithm [24–26].

### 2.3. Statistics

Demographics and patient characteristics were compared by the chi-square test, and the Kaplan-Meier method was used for survival analysis of outcomes. Living patients without second relapse at the time of analysis were censored at the date of last follow-up. The last survival and disease status update was performed in April 2022. The log-rank test was used for comparisons, and all data were analyzed by the Statistical Package for Social Sciences software, version 24.0 (IBM Corp, Armonk, NY, USA).

## 3. Results

### 3.1. Events after First-Line Treatment

**Figure 1:**
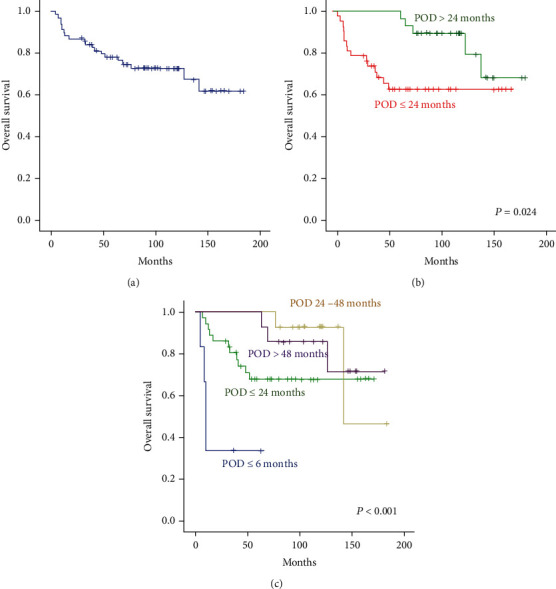
(a) Overall survival of 70 patients who experienced any kind of event. (b) Comparison of overall survival between patients with or without POD24. (c) Comparison of survival outcomes according to the time to first relapse or progression.**POD24**: Progression of disease within 24 months.

Seventy patients experienced an event during or after first-line treatment including RCVP (*n* = 35), RCHOP (*n* = 28), or BR (*n* = 7). At the time of first-line treatment, patients had a median age of 44.5 years (range: 29-79 years), and 38 were female. According to FLIPI at diagnosis, 25 patients belonged to the high-risk group (Table 1). At the time of first event, a greater number of patients had high-risk FLIPI scores than at time of diagnosis, and most patients had elevated serum LDH level (Table 1). Two patients were positive for HBsAg, 12 patients had anti-HBc antibodies, and all received prophylactic antiviral agents during and after treatment. Of 70 cases with relapsed or refractory FL, 42 patients experienced POD24, and six of these patients showed disease progression during first-line treatment. Large-cell transformation occurred in nine patients who showed POD24, whereas the remaining 28 patients who relapsed after 24 months did not have large-cell transformation. At the median follow-up of 104 months (95% CI: 90-118 months) after diagnosis, the median OS of the 70 patients who experienced any kind of event was not achieved (Figure 1(a)). However, 15 of 42 patients with POD24 died, whereas only 5 of 28 patients without POD24 died. Accordingly, POD24 patients experienced significantly worse OS than patients without POD24 (Figure 1(b)). Furthermore, the survival outcomes for patients were worst for those experiencing progression within six months after first-line treatment was started. Additionally, the OS of patients who relapsed four years after first-line treatment was not significantly different from that of patients who relapsed from 24 to 48 months (Figure 1(c)).

### 3.2. Comparison of Outcomes Based on First Salvage Treatment

**Figure 2:**
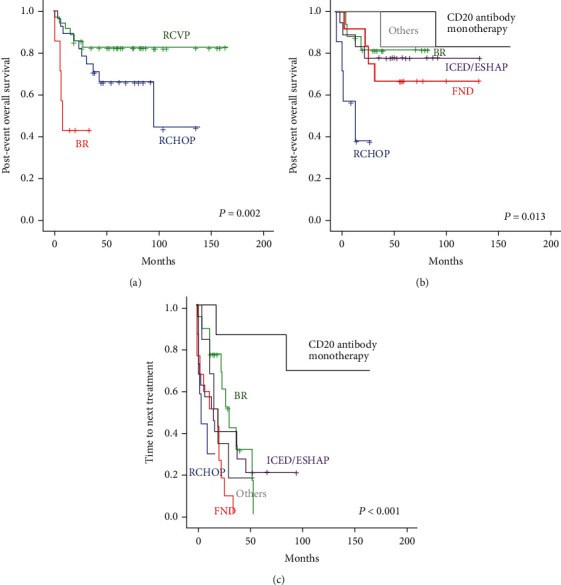
(a) Comparison of postevent overall survival according to type of first-line treatment. (b) Comparison of postevent overall survival according to type of salvage treatments after first relapse or progression. (c) Comparison of time to next treatment according to type of salvage treatments after first relapse or progression.

**Figure 3:**
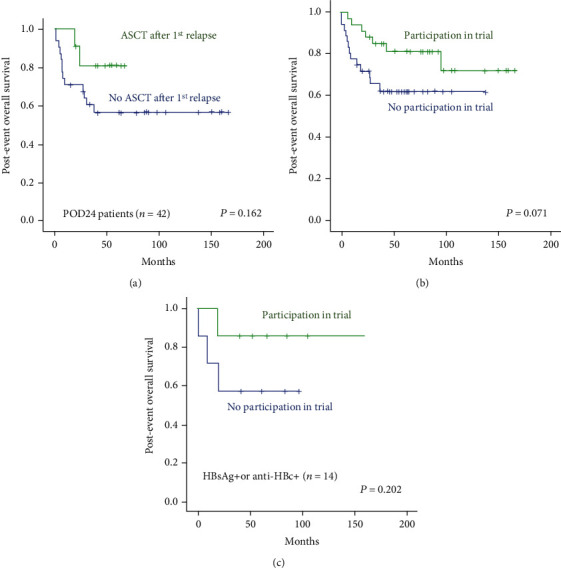
(a) Comparison of post-event overall survival by ASCT after first relapse or progression in patients with POD24. (b) Comparison of post-event overall survival by participation in clinical trials. (c) Comparison of post-event overall survival by participation in clinical trials in HBsAg-positive or anti-HBc antibody-positive patients.ASCT: Autologous stem cell transplantation; POD24: progression of disease within 24 months; HBsAg: hepatitis B surface antigen; anti-HBc antibody; hepatitis B core antibody.

Among 70 patients, most were initially treated with RCVP (*n* = 35) or RCHOP (*n* = 28), while only seven received BR (Table 1). The small number of patients with relapsed or refractory FL after BR was related to the better efficacy of BR as a first-line treatment, as we reported previously [27]. However, all patients who failed BR showed POD24 (7/7, 100%), whereas approximately half of patients who received RCVP (18/35, 51%) or RCHOP (17/28, 61%) showed POD24. Thus, once relapse occurred, postevent OS was worse in patients receiving BR than in patients who received RCVP or RCHOP (Figure 2(a)). Two patients died due to sepsis associated with febrile neutropenia that occurred after RCVP chemotherapy, and another patient developed B-acute lymphoblastic leukemia as a second malignancy during follow-up. After excluding those three patients, outcomes from subsequent salvage treatments after first relapse were analyzed. Salvage treatment types for first relapses were divided as follows: (1) BR, (2) RCHOP, (3) CD20-directed monotherapies such as rituximab and ofatumumab, (4) ICED (ifosfamide, carboplatin etoposide, and dexamethasone) or ESHAP (etoposide, cisplatin, cytarabine, and methylprednisolone), (5) FND (fludarabine, mitoxantrone, and dexamethasone), and (6) other treatments including PI3K (phosphoinositide 3-kinase) inhibitors or HDAC (histone deacetylase) inhibitors (Table 1). When postevent OS was compared by salvage treatment type, patients receiving RCHOP had the worst OS because they were mainly early relapsed patients after BR (Figure 2(b)). However, patients receiving BR, ICED/ESHAP, and FND also eventually showed relapse or progression, and 72% of patients (48/67) had secondary events requiring additional salvage treatments. The time to next treatment was similar among all patients except those receiving CD20-directed monotherapies such as rituximab and ofatumumab (Figure 2(c)). After the first event, ASCT was performed for 14 patients who responded to salvage chemotherapy. Of 42 patients with POD24, 11 underwent ASCT after first relapse, and their postevent OS was better than that of the 31 patients who did not receive ASCT after POD24, although the difference was not statistically significant (Figure 3(a)). During the treatment journey after first relapse, half of patients (34/67, 51%) participated in at least one clinical trial. When postevent OS was compared by participation in clinical trials, patients participating in at least one clinical trial, irrespective of treatment line, had better survival than patients who did not, although the difference was not statistically significant (Figure 3(b)). Seven of 14 HBsAg- or anti-HBc antibody-positive patients participated in clinical trials and had a better survival trend than patients who did not (Figure 3(c)).

### 3.3. Postevent Outcomes for Patients with Multiple Events

**Figure 4:**
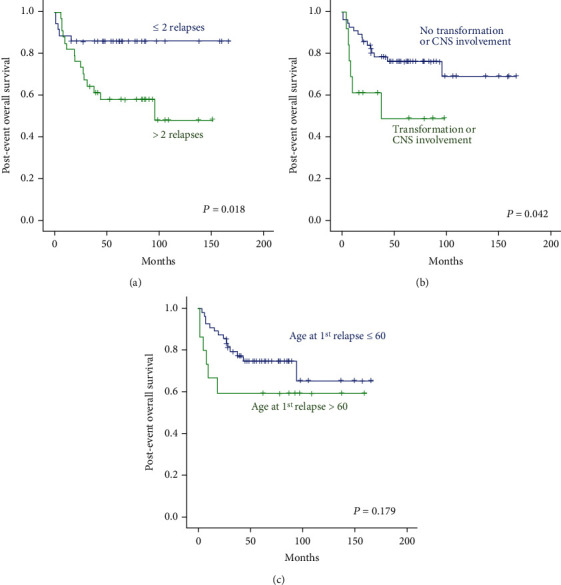
(a) Comparison of postevent overall survival according to number of relapses. (b) Comparison of postevent overall survival by occurrence of large-cell transformation or secondary central nervous system involvement. (c) Age at time of first relapse was not associated with postevent overall survival.

**Figure 5:**
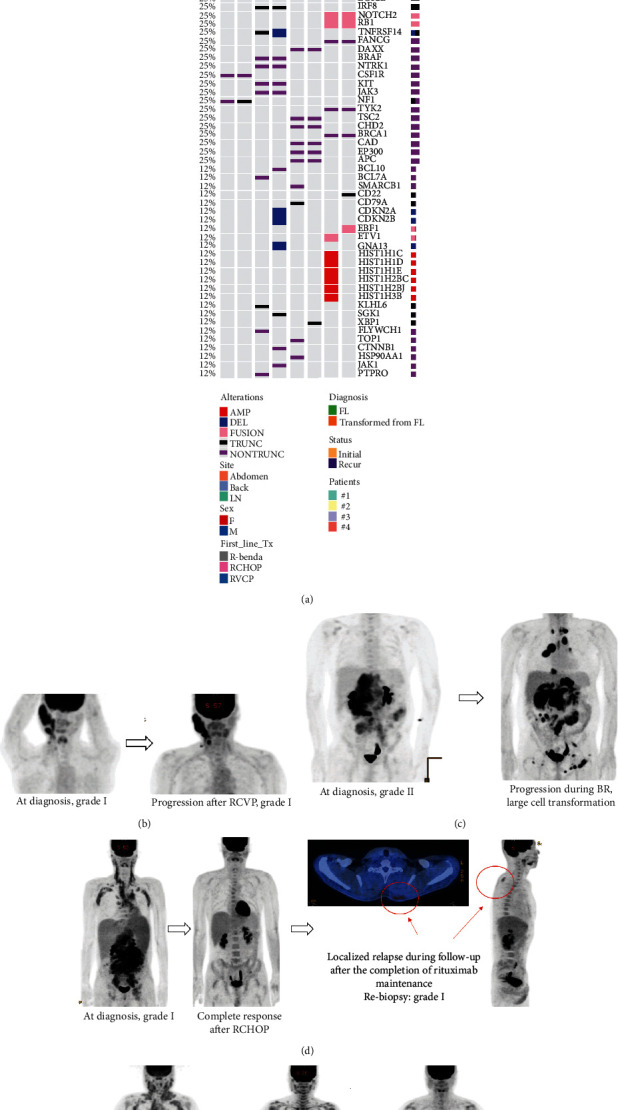
(a) Paired analysis of mutation profiles in four cases, including one case of large-cell transformation. (b) Case #1, a 57-year-old female showing persistence of a nodal lesion even after RCVP. (c) Case #2, a 39-year-old male with relapse immediately during BR and large-cell transformation refractory to subsequent salvage treatments. (d) Case #3, a 40-year-old male showing localized relapse with no systemic symptoms after RCHOP. (e) Case #4, a 49-year-old female who relapsed after RCHOP and was rescued by participating a clinical trial.

After 48 patients experienced a second relapse after their first salvage treatment, they were treated with additional salvage treatments. However, despite salvage treatment, 34 patients experienced more than two episodes of relapse or progression. Postevent overall survival was significantly worse in patients who experienced more than two relapses than in patients who did not (Figure 4(a)). During the clinical course, large-cell transformation was histologically confirmed in nine patients, and secondary central nervous system (CNS) involvement was found in five; postevent OS of these patients with large-cell transformation or secondary CNS involvement was significantly worse than in patients without it (Figure 4(b)). However, age at the time of first relapse was not associated with postevent overall survival, suggesting that patients older than 60 years could be rescued by subsequent treatments (Figure 4(c)). When we compared the mutation profiles from diagnosis with those from relapse or progression using paired tumor samples from four patients, truncated or nontruncated mutations were frequently found in CREBBP and BCL2 at the time of diagnosis and relapse (Figure 5(a)). In case #1, a 57-year-old female with grade I disease, stage III FL became refractory to RCVP (Figure 5(b)). However, rebiopsy of the lesion revealed grade I, and the comparison of mutation profiles demonstrated no additional mutations (Figure 5(a)). She received platinum-based ICED and maintained complete response after ASCT. In contrast, a 39-year-old male (case #2) progressing during BR showed large-cell transformation and additional changes including deletion of GNA13 (Figure 5(a) and 5(c)). He was refractory to subsequent salvage treatments and eventually died due to disease progression. A 40-year-old male (case #3) showed localized relapse with no systemic symptoms during follow-up after RCHOP and rituximab maintenance (Figure 5(d)). The rebiopsy of the lesion showed grade 1 and was same as that of his initial diagnosis, and the mutation profiles were not significantly different (Figure 5(a)). He was successfully managed with radiation therapy. A 49-year-old female with FL grade II (case #4) relapsed after RCHOP, and the rebiopsy showed grade II and no significant changes in mutation profile at the time of relapse (Figure 5(a)). Her disease status was rescued by participating in a trial with ibrutinib-containing chemotherapy (Figure 5(e)).

## 4. Discussion

In this study, we retrospectively analyzed survival outcomes of FL patients who relapsed or progressed after first-line treatment with rituximab-containing chemotherapy including RCHOP, RCVP, or BR to evaluate the impact of subsequent salvage therapies on prognosis. Although all patients experienced at least one event after their first-line treatment, OS did not reach the median value at the median follow-up of 104 months (95% CI: 90-118 months) after diagnosis, which highlights the indolent nature of FL (Figure 1(a)). Nevertheless, 15 of 42 patients who had an early event—so called POD24—died, whereas only five of 28 patients who relapsed after 24 months died. A recent pooled analysis of 13 clinical trials validated POD24 as a significant prognostic indicator for poor survival in FL patients, and our study also observed poorer survival outcomes for patients with POD24 regardless of type of frontline and salvage treatment (Figure 1(b)) [28]. Of 70 patients with relapsed or refractory FL in our study, the majority was treated with RCVP (*n* = 35) or RCHOP (*n* = 28) rather than BR (*n* = 7). However, although the absolute number of relapses or progressions was lower among patients receiving BR than RCVP or RCHOP, all patients who relapsed or progressed after BR had an early event. This was consistent with the relatively higher incidence of early progression during or after BR therapy in a population-based cohort of FL patients [29].

The BR regimen has been widely used as a frontline treatment for newly diagnosed FL patients due to its superior outcomes to RCVP and RCHOP in terms of progression-free survival and toxicity profiles [30, 31]. Accordingly, if FL patients were initially not treated with anthracycline-containing chemotherapy, they could be treated with CHOP-like regimens such as obinutuzumab-CHOP (GCHOP) [27]. However, our patients who initially received BR were treated with conventional chemotherapy such as ICED or FND because the GCHOP regimen was not available in Korea. Furthermore, RCHOP could be used only for patients who had large-cell transformation. Accordingly, postevent OS after the first relapse or progression was shortest in patients receiving RCHOP, while the outcomes for patients receiving BR, ICED, and FND were not significantly different (Figure 2(b)). Additionally, because most patients relapsed after these salvage treatments, the time to next treatment was not different except for patients receiving anti-CD20 monoclonal antibody alone such as rituximab or ofatumumab monotherapy (Figure 2(c)). The superior outcomes for patients receiving anti-CD20 monoclonal antibody alone were associated with the indolent nature of their tumors because rituximab or ofatumumab monotherapy was only used for patients with less aggressive symptoms and low tumor burden in our clinical practice.

For relapsed or refractory FL patients, particularly those with POD24, high-dose chemotherapy followed by ASCT has been proposed as a consolidative treatment [32]. However, ASCT could not be performed in all cases because patients who are candidates for this treatment are young, otherwise healthy, and should achieve complete or partial response after salvage therapy. Accordingly, not all patients underwent ASCT in our study, and only 14 underwent ASCT after their first relapse. Because the number of patients undergoing ASCT was relatively small, postevent OS of patients receiving ASCT was not significantly different from that of patients who did not receive it and who experienced POD24 (Figure 3(a)). However, given the trend of better survival outcomes in the ASCT group, consolidation treatment with ASCT could be considered for patients responding to salvage treatment, especially in cases of early relapse or progression. In our study, half of the patients (34/67, 51%) participated in at least one clinical trial during their treatment after first relapse. The patients participating in at least one clinical trial experienced better survival than those who did not, although the difference was not statistically significant (Figure 3(b)). However, this result could be due to selection bias because only patients with adequate organ function and performance status can be enrolled in clinical trials. Nevertheless, patients should always be recommended to participate in clinical trials because postevent OS and time to next treatment after conventional salvage chemotherapies were not satisfactory (Figure 2(b) and 2(c)). In B-cell lymphoid malignancies, in which anti-CD20 antibodies are essential treatment components, close to 10% of patients with positive HBsAg- or anti-HBc antibodies experienced reactivation of hepatitis B virus (HBV) [33]. Thus, HBsAg- or anti-HBc antibody-positive patients in general were excluded as candidates for clinical trials. In this study, participation in clinical trials was associated with better survival outcomes for even HBsAg- or anti-HBc antibody-positive patients although the sample size was small (Figure 3(c)). Considering that prophylactic antiviral agents and periodic HBV DNA monitoring are routinely performed in clinical practice, thoughtful consideration should be given to the potential inclusion of patients with HBV in clinical trials.

We conducted genomic analysis using paired tumor tissue samples at diagnosis and relapse and observed frequent occurrence of mutations in CREBBP and EZH2, which are involved in lymphomagenesis and progression as a chromatin regulator, as well as mutations in BNFRSF14 and BCL2 as previously reported [34]. Interestingly, the mutations observed at diagnosis and relapse were similar, although the number was too small for statistical analysis (Figure 4(c)). However, a case with large-cell transformation showed that occurrence of additional genomic alterations are consistent with worse survival outcomes (Figure 4(b)). In our study, patients who experienced repeated multiple relapses had worse survival outcomes than those who did not (Figure 4(a)). Furthermore, most patients eventually relapsed even after ASCT and other additional treatments including participation in clinical trials. Therefore, considering the final outcomes as well as quality of life of patients who experienced repeated relapses, more efficient treatment approaches are needed, such as chimeric antigen receptor (CAR) T-cells, for which favorable outcomes have been reported [35–37].

However, our study has some limitations as follows. First, the number of patients who were analyzed in this study was too small because FL is relatively uncommon in Korea compared to Western countries, and this was a single-center analysis. Accordingly, our survival analysis failed to reach statistically significant values in the comparison of survival outcomes based on various parameters such as participating in clinical trials and age at the time of first relapse. Furthermore, as our patients received various salvage treatments and had variable clinical conditions at the time of relapse or progression, there were many factors potentially influencing survival outcome after relapse or progression. That was why we could not conduct multivariate analysis for identification of risk factors predicting outcome of FL patients after relapse. Thus, further study with larger population should be warranted to establish a prognostic model for relapsed or refractory FL patients. Second, due to the retrospective nature of this study, several clinically important issues could not be addressed such as the occurrence of deep vein thrombosis (DVT) and pulmonary thromboembolism (PE) although FL patients might be at risk of DVT and PE in clinical practice [38, 39]. In addition, most patients received various salvage treatments including participation in clinical trials. Thus, there might be some potential effects of drug-drug interactions on the outcome of patients as previously reported [40, 41]. However, those issue could not be addressed, either because there were no data available for analyzing them. Given the importance of those factors, further more detailed studies also might be required.

In conclusion, although the sample size of our study was relatively small, we evaluated patient survival outcomes after relapse or progression during or after first-line treatment. Due to the poor outcomes of patients with POD24, high-dose chemotherapy followed by ASCT should be considered for patients with chemotherapy-sensitive relapse. Additionally, participation in clinical trials should be considered during any point in the clinical course of relapse or refractory FL. However, given the unmet needs of relapsed or refractory FL patients, novel therapies such as CAR T-cells should be more actively used to improve outcomes of FL patients in terms of relapse prevention.

## Figures and Tables

**Table 1:** Patient characteristics.

Parameters		At diagnosis	At first event
Age	Median (range), years	44.5 (29–79)	52.0 (30–80)
	≤60	60 (86%)	55 (79%)
	>60	10 (14%)	15 (21%)
ECOG performance status	0/1	65 (93%)	65 (93%)
	≥2	5 (7%)	5 (7%)
Ann Arbor stage	I/II	7 (10%)	6 (9%)
	III/IV	63 (90%)	64 (91%)
FLIPI	Low risk (0–1)	19 (27%)	0 (0%)
	Intermediate risk (2)	26 (37%)	17 (24%)
	High risk (3–5)	25 (36%)	53 (76%)
Histologic grade	1	41 (59%)	33 (47%)
	2	16 (23%)	11 (16%)
	3A	13 (18%)	17 (24%)
	Large cell transformation	—	9 (13%)
Number of nodal involvements	≥5	36 (51%)	42 (60%)
	<5	34 (49%)	28 (40%)
Bone marrow involvement	Presence	43 (61%)	Unknown
B-symptoms	Presence	4 (6%)	3 (4%)
Elevated LDH	Presence	19 (27%)	67 (96%)
Treatment	RCVP	35 (50%)	—
	RCHOP	28 (40%)	7 (10%)
	BR	7 (10%)	17 (24%)
	Anti-CD20 antibody monotherapy	—	7 (10%)
	ICED/ESHAP	—	18 (26%)
	FND	—	12 (17%)
	Other treatments	—	6 (9%)
	Not applicable	—	3 (4%)

ECOG: eastern cooperative oncology group; FLIPI: follicular lymphoma international prognostic index; LDH: lactate dehydrogenase; RCVP: rituximab, cyclophosphamide, vincristine, and prednisone; RCHOP: rituximab, cyclophosphamide, vincristine, prednisone, and doxorubicin; BR: bendamustine and rituximab; ICED: ifosfamide, carboplatin, etoposide, and dexamethasone; ESHAP: etoposide, cisplatin, cytarabine, and methylprednisolone; FND: fludarabine, mitoxantrone, and dexamethasone.

## Data Availability

All of the data analyzed are included in this published article. The datasets collected during the study are available from the corresponding author on reasonable request.
